# Use of hierarchical models to evaluate performance of cardiac surgery centres in the Italian CABG outcome study

**DOI:** 10.1186/1471-2288-7-29

**Published:** 2007-07-03

**Authors:** Paola D'Errigo, Maria E Tosti, Danilo Fusco, Carlo A Perucci, Fulvia Seccareccia, the research group of the Italian CABG Outcome Study

**Affiliations:** 1 National Center for Epidemiology, Surveillance and Health Promotion, Istituto Superiore di Sanità, Rome, Italy; 2 Department of Epidemiology, ASL RME, Rome, Italy; 3 See Appendix

## Abstract

**Background:**

Hierarchical modelling represents a statistical method used to analyze nested data, as those concerning patients afferent to different hospitals. Aim of this paper is to build a hierarchical regression model using data from the "Italian CABG outcome study" in order to evaluate the amount of differences in adjusted mortality rates attributable to differences between centres.

**Methods:**

The study population consists of all adult patients undergoing an isolated CABG between 2002–2004 in the 64 participating cardiac surgery centres.

A risk adjustment model was developed using a classical single-level regression. In the multilevel approach, the variable "clinical-centre" was employed as a group-level identifier. The intraclass correlation coefficient was used to estimate the proportion of variability in mortality between groups. Group-level residuals were adopted to evaluate the effect of clinical centre on mortality and to compare hospitals performance. Spearman correlation coefficient of ranks (*ρ*) was used to compare results from classical and hierarchical model.

**Results:**

The study population was made of 34,310 subjects (mortality rate = 2.61%; range 0.33–7.63). The multilevel model estimated that 10.1% of total variability in mortality was explained by differences between centres. The analysis of group-level residuals highlighted 3 centres (VS 8 in the classical methodology) with estimated mortality rates lower than the mean and 11 centres (VS 7) with rates significantly higher. Results from the two methodologies were comparable (*ρ *= 0.99).

**Conclusion:**

Despite known individual risk-factors were accounted for in the single-level model, the high variability explained by the variable "clinical-centre" states its importance in predicting 30-day mortality after CABG.

## Background

Over the last decade, worldwide interest in using "outcome studies" to evaluate performance of health services has been increasing [1-6].

In Italy, the first outcome evaluation at the national level began in 2002 with the "Italian coronary artery bypass graft surgery (CABG) outcome study", a prospective voluntary study on short-term outcomes in patients undergoing CABG surgery [7]. The aim was to provide comparable data on observed and expected mortality 30 days after CABG intervention in each cardiac surgery centre.

Thirty-day mortality is recognized as a good indicator of the quality of care in cardio-thoracic surgery. However, in order to accurately measure performance, mortality should be adjusted for pre-existing clinical conditions.

Comparative data, especially if adjusted using a risk function empirically derived from the observed population, serve many purposes and have the potential to provide insight and improve the quality of care. Unfortunately, the existing standard single-level models, usually adopted in outcome studies, treat all patients as independent observations and ignore that they are grouped within hospitals. Patients undergoing a surgical intervention within the same hospital may be correlated, violating one of the basic assumptions of traditional regression analysis. Hierarchical (or multilevel) models consider the hospitals involved in the study as a random sample from a population of hospitals and partition the random variability of data into variability between different patients and between different hospitals. The hospital-specific random error component is interpreted as representing differences in hospital quality. Consequently, hierarchical modelling is strongly advocated as a more appropriate statistical method for dealing with outcomes data when individual patients are clustered within hospitals [8-12]. Moreover, hierarchical models account for regression-to-the mean by providing estimates of standardized mortality rates that are appropriately less extreme than the observed ones. Hospitals with small sample sizes are more likely to have extreme observed mortality rates because of chance variation, their true rates usually being less extreme than their observed. Estimates from hierarchical models provide more accurate assessments, with the most improvement for smaller hospitals because they experience greater regression-to-the mean [11,13-15].

Presently, the use of hierarchical models to rank clinical centres' performance still represents a relatively new statistical approach, not widely employed, but that certainly deserves to be tested.

The aim of this paper is to build a hierarchical regression model using data from the "Italian CABG outcome study" in order to evaluate the amount of differences in adjusted mortality rates attributable to differences between centres.

## Methods

### Study Population

In the "Italian CABG outcome study" 82 of 89 existing cardiac surgery centres agreed to participate.

Between January 1^st ^2002 and September 30^th ^2004 any patient aged 15–99 years who underwent an isolated CABG surgery (not associated with other cardiac or extra-cardiac procedures) at one of the participating centres was considered eligible for the study.

Only centres that met specific inclusion criteria reported in the study protocol were included in the analytical database [7].

### Individual-level variables (patients' characteristics)

For each patient enrolled the centres collected a set of demographic variables (gender, age, residence, and place of birth) and clinical characteristics (diabetes under treatment, malignant ventricular arrhythmia, cirrhosis, chronic obstructive pulmonary disease, renal failure, neurological dysfunction, active endocarditis, pulmonary hypertension, cancer, extra-cardiac arteriopathy and stroke, haemodynamic condition, ventricular dysfunction, previous surgery that opened the pericardium, unstable angina and recent infarction). Information on the type and circumstances of the intervention (CABG isolated intervention, associated with other cardiac or extra-cardiac procedures, elective or emergency, on-pump or off-pump circulation) was also collected. An active follow-up to determine patients' life status was carried out. In case of death within 30 days of the intervention, date and specific cause were recorded. The definitions of variables as well as a detailed description of methods have been published previously [7].

### Group-level variables (Surgery centres characteristics)

Simultaneous to the conduction of the "Italian CABG outcome study" was a survey on the "Italian cardiac surgery centres characteristics" carried out by the Italian Society of Cardiac Surgery.

The following information was selected to characterize surgery centres and used as "group-level variables": teaching/non-teaching medical facility, presence of an emergency department, presence of intensive care department, total number of available operating theatres, presence of operating theatres used only by cardiac surgery, number of nurses, number of beds, annual number of procedures, and percentage of coronary surgery. In addition, official data from the Italian Ministry of Health provided information on annual volume of CABG interventions performed at each centre.

### Statistical methods

Univariate analyses were used to compute crude odds ratios for all potential confounding factors. We identified the best risk adjustment model using a single-level multiple logistic regression to account for joint confounding. The potential predictive variables were selected using a conventional stepwise method with a cross validation procedure. First, all possible confounding variables were included in the model. Second, a backward stepwise method was used in order to identify independent associations with the outcome. Patients were randomly split into two equal-size samples: sample I was used to build the predictive model (n = 17,231); sample II was used as an independent database for model validation (n = 17,079). The entire data set was finally used to estimate the definitive coefficients and calculate their p-values, in order to provide more precise parameter estimates. A set of biologically plausible interaction hypotheses defined "a priori" was also tested (gender and age with variables identifying each hospital) [7].

To assess the calibration (accuracy) of the risk adjustment function obtained, the Hosmer-Lemeshow chi-squared test was applied. To evaluate the model's discriminative ability to predict individual deaths, the area under the Receiver Operating Curves (ROC) was measured.

Each patient's predicted probability of death was obtained using standard logistic regression. Expected number of deaths for each hospital was obtained by summing the predicted probabilities of death for all of the patients treated in that hospital. This number was compared with the observed number of death by taking the ratio observed/expected death and multiplying by the statewide mortality rate to obtained the Risk Adjusted Mortality Rate (RAMR). Hospitals with RAMRs that were significantly higher or lower than the statewide rate were identified as high outliers or low outliers, respectively. The exact method was used to identify hospitals significantly different from the mean [16]. RAMRs were ordered to obtain centres' ranking based on classical logistic model.

After the best set of individual-level confounders was identified, the variable that define the clusters ("clinical centre"), was introduced to test the suitability of a multilevel model [10,11]. This approach examines the effect of group-level and individual-level variables on individual-level outcome (30-day mortality) simultaneously.

The model used was a mixed logit model with random intercepts and fixed slopes [17]. The assumption is that the covariates' effects are fixed among the Centres, whereas the mean effect of each hospital is allowed to vary. The maximum likelihood estimates of this model have been obtained by adaptive quadrature [18].

A Likelihood Ratio (LR) test was used to evaluate the effect of both the group-level variable (single-level versus multilevel logistic regression) and the group-level covariates; the latter were tested one at a time. When the LR test was not applicable, the Wald test was used.

We compared the SE/Coefficient ratios from both single-level and multilevel logistic regression to check for possible bias in those from the single-level model (erroneously small SE).

We used the "intraclass correlation coefficient" (ICC) to estimate the variability in mortality between groups; this coefficient represents the proportion of variability explained by the presence of clusters in the observed population [12,19].

The approximate ICC by Snijders and Bosker was used:

ICC=τ0τ0+π2/3
 MathType@MTEF@5@5@+=feaafiart1ev1aaatCvAUfKttLearuWrP9MDH5MBPbIqV92AaeXatLxBI9gBaebbnrfifHhDYfgasaacH8akY=wiFfYdH8Gipec8Eeeu0xXdbba9frFj0=OqFfea0dXdd9vqai=hGuQ8kuc9pgc9s8qqaq=dirpe0xb9q8qiLsFr0=vr0=vr0dc8meaabaqaciaacaGaaeqabaqabeGadaaakeaacqWGjbqscqWGdbWqcqWGdbWqcqGH9aqpdaWcaaqaaGGaciab=r8a0naaBaaaleaacqaIWaamaeqaaaGcbaGae8hXdq3aaSbaaSqaaiabicdaWaqabaGccqGHRaWkcqWFapaCdaahaaWcbeqaaiabikdaYaaakiabc+caViabiodaZaaaaaa@3C6C@

were *τ*_0 _is the estimated variance of the random effect of hospital on the mean and *π *is the quantity 3.14159 [15].

Differently from the classical approach, the hierarchical model identifies the outliers using the group-level residuals. Trying to simplify the concept, it may be stated that the group-level residual, for each centre, represents the distance between its estimated mortality and the overall estimated mortality.

In order to compare clinical centres performances with the overall mean, group-level residuals were ordered from the smallest to the largest and graphically presented together with their 95% confidence intervals (CI) [17,20-22].

Centres with residuals significantly less than zero (CI not overlapping the zero row) performed better than the overall population, whereas centres with residuals significantly higher than zero performed worse [20-22]. Spearman correlation coefficient of ranks and a scatter-plot were used to compare results from standard logistic regression with those obtained by the hierarchical model.

All the statistical procedures were performed using STATA 8.1 statistical software.

## Results

Out of the 82 participating centres, 12 were excluded because more than 5% of patients were lost to follow-up, two centres collected data for less than six months, and four centres were excluded because they performed fewer than 100 CABG interventions per year. Sixty-four centres from 20 Italian regions met all the inclusion criteria.

During the study period in the 64 cardiac surgery centres, 34,611 subjects underwent an isolated CABG intervention and were enrolled. Three hundred and one subjects (0.87%) were lost to follow-up thus reducing the study population to 34,310 subjects. The median number of patients enrolled at each centre was 470 (range 116 – 2,058).

Table 1 shows the characteristics of the study population: the mean age was 67.4 (SD: 9.40), 19.4% of subjects were 70 years or older and almost 80% of population was male. The most frequently reported comorbidities were: recent infarction, diabetes, unstable angina and extra-cardiac arteriopathy. The ejection fraction was = 50 for about 71% of the patients, but was < 30 for 2.75%; 2.23% of subjects had undergone a previous CABG; during the intervention 29.4% of patients had off pump circulation.

**Table 1:** Characteristics of the study population and odds ratios for 30-day mortality estimated by univariate models

	**Mean**	**Standard Deviation**	**Odds Ratio**	**95% CI**
**Age**	67.4	9.40	1.07	1.06 – 1.08
				

	**Cases**	**%**	**Odds Ratio**	**95% CI**

**Gender (fem) **	7143	20.90	1.46	1.26 – 1.70
**Shock**	361	1.06	14.44	11.29 – 18.47
**Unstable haemodynamic **	2681	7.90	4.01	3.42 – 4.70
**condition before surgery**				
**Diabetes**	9600	28.00	1.44	1.26 – 1.66
**Malignant ventricular **	566	1.66	3.53	2.60 – 4.79
**Arrhythmia**				
**Cirrhosis**	141	0.41	2.86	1.50 – 5.46
**Chronic obstructive**	3460	10.10	2.25	1.90 – 2.66
**pulmonary disease**	352	1.03	6.66	4.91 – 9.04
**Dialysis**				
**Serum creatinine>2 mg/dl**	1278	3.73	4.60	3.78 – 5.60
**Neurological dysfunction**	804	2.46	1.73	1.23 – 2.45
**Endocarditis**	54	0.16	2.21	0.69 – 7.08
**Pulmonary hypertension**	114	0.34	6.29	3.69 – 10.71
**Cancer**	434	1.27	1.82	1.15 – 2.86
**Extra-cardiac arteriopathy**	7304	21.30	2.44	2.13 – 2.80
**Stroke**	1335	3.90	1.99	1.54 – 2.58
**Ejection fraction**				
**≥ 50**	23772	71.17	*Reference*	
**30–49**	8713	26.10	2.49	2.16 – 2.87
**<30**	917	2.75	7.23	5.76 – 9.07
**missing**	908	2.65	1.22	0.77 – 1.94
**Emergency**	1311	3.83	7.22	6.07 – 8.59
**Previous CABG**	763	2.23	2.96	2.23 – 3.94
**Any other previous surgery that opened the pericardium**	403	1.17	1.97	1.25 – 3.10
**Unstable angina**	8387	24.40	2.63	2.3 – 3.01
**Recent infarction**	9615	28.00	1.76	1.54 – 2.02
**Off pump circulation**	10073	29.40	0.98	0.85 – 1.14

Thirty days after the CABG intervention, 895 patients had died, with a crude mortality rate of 2.61%. There was great variability observed in crude mortality rates among centres (range 0.33–7.63).

The factors most strongly associated with the outcome as identified by the univariate analysis were: ejection fraction, dialysis, pulmonary hypertension, shock and emergency condition; factors not significantly associated were endocarditis and the "on/off pump circulation" (Table 1).

The model did not select the following factors to be included: unstable haemodynamic condition before surgery, cirrhosis, neurological dysfunction, endocarditis, cancer, stroke, any other previous surgery that opened the pericardium, and the use of off pump circulation. The selected model when applied to the whole population performed well: the Hosmer-Lemeshow statistic was 18.08 (p = 0.35) and the area under the ROC curve was 0.80.

Table 2 reports the risk factors selected for the model with their estimated odds ratios (OR) and 95% confidence intervals (CI). The single-level logistic model confirmed that the following factors are the ones most strongly associated with 30 day mortality after CABG surgery: emergency (OR = 3.89, CI = 3.12 – 4.85), shock (OR = 3.44, CI = 2.48 – 4.78), dialysis (OR=3.41,   CI=2.36-4.93), pulmonary hypertension (OR=2.26, CI=1.16-4.40), and ejection fraction < 30 (OR = 3.14, CI = 2.35 – 4.20). A strong association was also found for previous CABG intervention (OR = 2.86, CI = 2.10 – 3.89).

**Table 2:** Risk factors for 30-day mortality: odds ratios by the single-level and the multilevel logistic model

	**Single-level logistic model**		**Multilevel logistic model**	
**Patient Risk Factors**	**Odds Ratio**	**95% CI**	**Odds Ratio**	**95% CI**

**Age**	0.96	0.87 – 1.05	0.95	0.87 – 1.04
**Age**^2^	1.00	1.00 – 1.00	1.00	1.00 – 1.00
**Gender (fem)**	1.29	1.09 – 1.52	1.25	1.06 – 1.48
**Shock**	3.44	2.48 – 4.78	4.02	2.85 – 5.68
**Diabetes**	1.35	1.16 – 1.58	1.32	1.13 – 1.54
**Dialysis**	3.41	2.36 – 4.93	3.29	2.27 – 4.79
**Pulmonary hypertension**	2.26	1.16 – 4.40	1.90	0.97 – 3.73
**Malignant ventricular arrhythmia**	1.46	1.01 – 2.12	1.44	0.98 – 2.13
**Chronic obstructive pulmonary disease**	1.52	1.26 – 1.84	1.51	1.24 – 1.84
**Serum creatinine>2 mg/dl**	2.08	1.63 – 2.65	2.38	1.85 – 3.05
**Extra-cardiac arteriopathy**	1.72	1.48 – 2.01	1.89	1.61 – 2.21
**Unstable angina **	1.53	1.31 – 1.79	1.58	1.34 – 1.86
**Previous CABG**	2.86	2.10 – 3.89	3.21	2.34 – 4.41
**Emergency**	3.89	3.12 – 4.85	4.19	3.31 – 5.29
**Ejection fraction (vs ≥ 50)**				
**30–49**	1.80	1.54 – 2.10	1.83	1.56 – 2.14
**<30**	3.14	2.35 – 4.20	3.56	2.65 – 4.78
**missing**	1.36	0.75 – 2.45	1.41	0.74 – 2.67

The variables selected by the single-level logistic model were then included in a multilevel model. The LR test used to compare the single with the multilevel model showed a very significant improvement when the group-level variable ("clinical centre") was introduced (p < 0.001).

The previously identified group-level covariates were tested, but the LR test did not show significant improvement. Group-level covariates were also tested using a classical single-level logistic model, but no significant contribution was found.

The best model was the sparest hierarchical model with no group-level covariates that estimated an ICC = 0,101. This means that 10.1% of the total variability is explained by differences between cardiac surgery centres.

Table 2 lists the ORs estimated with the hierarchical model for individual-level covariates. The ORs and 95% CI of the single-level and multilevel logistic regression estimates were only slightly different.

Coefficients, SE and SE/Coefficient ratios for the risk factors introduced in both models are reported in Table 3. As expected, SE/Coefficient ratios from the multilevel model in most cases are higher than the others.

**Table 3:** Coefficients, Standard Errors (SE), SE/Coefficient ratios estimated by the single-level and the multilevel logistic model

	**Single-level logistic model**			**Multilevel logistic model**		
**Patient Risk Factors**	**Coefficients**	**SE**	**SE/Coeff.**	**Coefficients**	**SE**	**SE/Coeff.**

**Age**	-0.04	0.05	-1.25	-0.05	0.04	-0.80
**Age**^2^	0.00	0.00	-	0.00	0.00	-
**Gender (fem)**	0.26	0.11	0.42	0.23	0.11	0.48
**Shock**	1.24	0.58	0.47	1.39	0.71	0.51
**Diabetes**	0.30	0.10	0.33	0.28	0.10	0.36
**Dialysis**	1.23	0.64	0.52	1.19	0.63	0.53
**Pulmonary hypertension**	0.82	0.77	0.94	0.64	0.65	1.02
**Malignant ventricular arrhythmia**	0.38	0.28	0.74	0.37	0.29	0.78
**Chronic obstructive pulmonary disease**	0.42	0.15	0.36	0.41	0.15	0.37
**Serum creatinine>2 mg/dl**	0.73	0.26	0.36	0.87	0.30	0.34
**Extra-cardiac arteriopathy**	0.54	0.13	0.24	0.64	0.15	0.23
**Unstable angina **	0.43	0.12	0.28	0.46	0.13	0.28
**Previous CABG**	1.05	0.45	0.43	1.17	0.52	0.44
**Emergency**	1.36	0.44	0.32	1.43	0.50	0.35
**Ejection fraction (vs ≥ 50)**						
**30–49**	0.59	0.14	0.24	0.60	0.15	0.25
**<30**	1.15	0.46	0.40	1.27	0.54	0.43
**missing**	0.30	0.41	1.37	0.34	0.46	1.35

**Figure 1:**
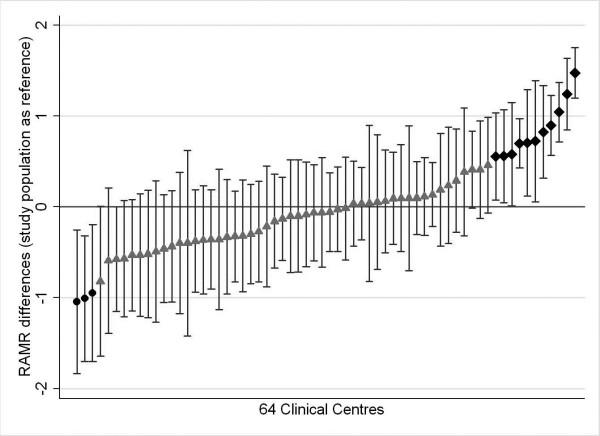
**Second level residuals and their confidence intervals, obtained by the multilevel model. **The black points represent those Centres which residual is significantly lower than zero, the black rhombs stand for the Centres with a significantly higher residual, the grey triangles represent Centres that show a residual not significantly different from zero.

Second-level residuals, obtained by the hierarchical model, were used to evaluate the effect of each clinical centre and are presented in figure 1 with their confidence intervals. In particular, 3 of the 64 centres analysed (4.7%) had a residual significantly lower than zero (RAMR ranging from 0.28 to 0.73) and 11 centres (17.2%) significantly higher (RAMR ranging from 5.25 to 10.44); 50 centres (78.1%) showed a residual not significantly different from zero. Using the classical approach, estimated mortality rates were found to be significantly lower than the mean (RAMR ranging from 0.26 to 1.32) in 8 centres and significantly higher (RAMR ranging from 4.37 to 8.76) in 7 centres. The hierarchical model confirmed that 3 centres identified by the classical approach as low outliers performed better than the mean. On the contrary, 4 centres among the high outliers identified by the hierarchical model were found to perform not differently from the mean using the classical model.

The use of second-level residuals and their confidence intervals allowed the 64 surgery centres to be divided into three categories, based on performance. There were no statistically significant differences in the group-level covariates in these three categories.

**Figure 2:**
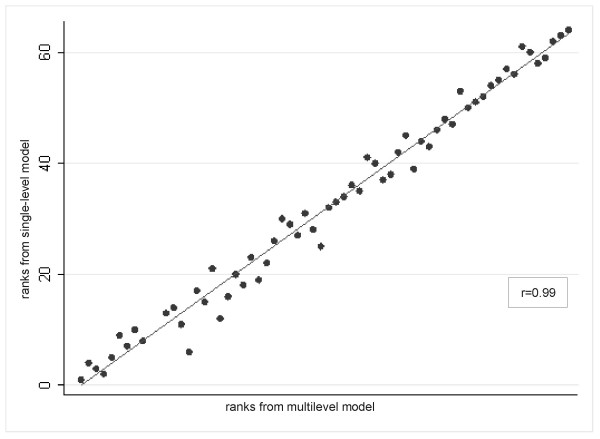
Scatter-plot of ranks obtained by the multilevel and the single-level logistic regression and Spearman Correlation coefficient.

The centres' ranking obtained using this analysis compared with that obtained by classical logistic regression had a correlation coefficient of 0.99 (Figure 2).

## Discussion

This study was conducted to build a hierarchical logistic model using data from the "Italian CABG outcome study".

The Italian CABG outcome study collected data from 2002 to 2004. Thirty-day overall mortality was 2.61%, comparable to the mortality observed by similar studies in other countries [5,6,29-31], but with a great variability among surgery centres (range 0.33 – 7.63). To investigate this heterogeneity, an empirical algorithm with a single-level multiple logistic regression procedure was used [7].

Standard single-level models, usually adopted in these kinds of studies, treat all patients as independent observations, when developing the risk-adjustment equation. Actually, patients undergoing a surgical intervention are not randomly allocated but nested in hospitals on the basis of reasons that lead them to make the same choices (place of residence, trust in a particular surgeon's skill, hospital's reputation, etc), thus violating one of the basic assumptions of traditional regression analysis. Hierarchical (or multilevel) models consider the hospitals involved in the study as a random sample from a population of hospitals and partition the random variability of data into two parts: that between different patients and that between different hospitals. The hospital-specific random error component is interpreted as representing differences in hospital quality.

Moreover, hierarchical models account for regression-to-the mean by providing more accurate assessments of standardized mortality rates, giving more robust estimates to small sample sizes, with the most improvement for smaller centres [11-14].

Therefore, hierarchical modelling represents the most appropriate statistical method for dealing with outcomes data when individual patients are clustered within hospitals and, in particular, when there is a great heterogeneity in sample size [8-14].

In spite of these characteristics, the scientific literature still lacks outcome studies that have actually employed the hierarchical methodology [20,23-27]. One possible cause could be represented by the high level of technology (software and hardware) required to implement the multilevel methodology [28], but the most convincing reason is the well-known risk of under/overestimating the importance of clinical centres in determining the outcome variability if the adjustment for confounding factors is not exhaustive.

In the single-level model, built on the Italian CABG study data, both demographic variables and comorbidities, recognized as important risk factors, were used to adjust outcome estimates. Therefore, the assumption was that any residual differences in outcome between centres should only reflect differences in quality of care. The same algorithm was used to build a hierarchical logistic model. The effects of patient characteristics on outcome (coefficients of factors) are comparable using both the single and the multilevel models, but multilevel SEs result greater than the others.

As other authors have underlined, hierarchical regression models could result in different RAMR from that obtained using conventional logistic regression, even though only patient-level characteristics are used in both models [32]. Actually, in this work some negligible differences between RAMRs obtained by the single and the multilevel approaches were found, but the overall findings of the study remain comparable.

The multilevel analysis showed that 10.1% (ICC) of the differences in the adjusted mortality rates were attributable to differences between centres. This amount of variability explained by the group-level variable is higher than that reported in other studies. Hannan et al, by applying a multilevel model on New York State CABG Registry data, found a percentage of variability not higher than 3.6%, attributable to the hierarchy and indicating only slight intraclass correlation [18]. The 10.1% of variability identified in this work seems to be more similar to the 12.6% identified by Austin et al. in their work on Myocardial Infarction (American Heart Journal, 2003) [32].

Considering that in this study all known individual factors associated with the outcome had already been accounted for in the single-level risk-adjustment model, the high value of the ICC indicates the great importance of cardiac surgery centres, *per se*, in predicting 30-day mortality after CABG intervention in the Italian context. This large amount of variability explained by the second level variable could be attributable to some unknown, or not-directly measurable characteristics (such as surgeon's ability), or to a "pool of unfavourable characteristics", whose effects are negligible if considered individually.

Results from the analysis of group-level residuals allowed us to divide the centres into three groups based on their performances (better, equal or worse than the mean). The three groups did not differ with regard to any of the group-level covariates considered; this finding was expected since those covariates were tested and then excluded from the final multilevel model.

The hierarchical model confirmed findings from the classical methodology. In fact, when results from the classical logistic regression were compared with those from the multilevel analysis, the two computed centres' ranking resulted very similar (correlation coefficient 0.99). However, accounting for clustering of observations, the hierarchical regression model provides truthful estimates of SE for the confounding factors and assures less biased results.

Concerning low outliers, results from this analysis in identifying fewer outliers using a multilevel rather than a classical single-level approach are in line with those from other published works [28]. This is not valid for high outliers, may be because in this study theyalso represent the highest volume hospitals. In fact, in this last case, the number of high outliers does not decrease since the effect of the regression-to-mean bias is negligible and not able to reducestatistical significance when comparing centres with the overall mortality.

### Study limitations

It is important to highlight that we should be cautious in interpreting the ratio of the between-group and total variation (ICC): if some important individual-level covariates were omitted from the single-level model, the ratio could overestimate the amount of variation between groups, thus attributing undue importance on the clinical centre. In our study all individual-level covariates known to be important were tested, but the possibility that other unidentified characteristics may be relevant cannot be excluded. On the other hand, the omission of a few group-level covariates, unknown as potential confounders, may have overstated the contribution of the individual-level factors.

Moreover, it should be stressed that the information on hospital characteristics was gathered for other purposes than this study, and a proper quality control of data collection could not be assessed. On the other hand these characteristics are sometimes difficult to survey within the Italian system, and routine procedures able to detect them have not yet been implemented at the national level. Other studies, trying to develop new and valid instruments that can better measure cardiac surgery centre characteristics, would be of great interest and could contribute to warrant a more appropriate use of hierarchical methodologies in this field.

## Conclusion

In the Italian CABG outcome study, a large amount (10%) of the differences in the adjusted mortality rates is attributable to differences between centres.

In spite of this finding, hospitals' ranking from hierarchical model almost completely overlapped that obtained from classical methodology [7].

This work can contribute to the debate by offering a rare example of an application of multilevel models to the evaluation of hospitals' performance.

## Competing interests

The author(s) declare that they have no competing interests.

## Authors' contributions

PD and MET participated in the design of the study, performed the statistical analysis and helped to draft the manuscript. DF performed the statistical analysis and helped to draft the manuscript. CAP and FS conceived of the study, participated in its design and coordination and helped to draft the manuscript. All authors read and approved the final manuscript.

## Appendix

### Research Group of the "Italian CABG Outcome Study"

Greco D, Italian Ministry of Health, Rome, Italy

Seccareccia F, D'Errigo P, Tosti ME, Rosato S, Badoni G, Manno V; National Centre of Epidemiology, Surveillance and Health Promotion – Istituto Superiore di Sanità, Rome, Italy

Perucci CA, Fusco, D Arcà M; Department of Epidemiology – ASL RME, Rome, Italy

### Scientific Committee

Di Eusanio G, "Lancisi" Cardiologic Institute, Ancona, Italy

Greco D, Italian Ministry of Health, Rome, Italy

Grilli R, Regional Health Authority Agency, Bologna, Italy

Pasini E, (representative of the Federation of Medical-Scientific Societies) "S. Maugeri" Foundation, Gussago, BS, Italy

Perucci CA, Department of Epidemiology – ASL RME, Rome, Italy

Rebuzzi AG, Cardiologic Institute, Policlinico "A. Gemelli", Rome, Italy.

Spolaore P, Regional Health Authority Agency, Castelfranco Veneto, TV, Italy

Taioli E, "Policlinico IRCCS" Hospital, Milan, Italy

Turinetto B, "Hesperia Hospital", Modena, Italy

## Pre-publication history

The pre-publication history for this paper can be accessed here:


